# Burnout syndrome in the Czech Republic: The decreasing trend over the years

**DOI:** 10.3389/fpubh.2023.1099528

**Published:** 2023-04-06

**Authors:** Martina Sebalo Vňuková, Ivan Sebalo, Tibor Brečka, Martin Anders, Radek Ptáček

**Affiliations:** ^1^Department of Psychiatry, Charles University and General University Hospital, Prague, Czechia; ^2^Centre of Research and Education in Forensic Psychology, School of Psychology, University of Kent, Canterbury, United Kingdom

**Keywords:** burnout, awareness, SMBM, Czech, adult

## Abstract

**Introduction:**

Burnout syndrome is a state of long-term work exhaustion that manifests on three levels: cognitive, physical and emotional. Research regarding burnout syndrome has spiked in recent years. Despite burnout syndrome not being a clinical diagnosis, it has been recognized as a significant reason for work absence or, in some cases, even work leave. This study examines trends in burnout in the Czech population. The main aim of this research was to fill in the burnout literature gap and document the burnout trend over the years. Our secondary aim was to see if there is awareness regarding burnout syndrome and whether, over the years, we will see an increasing or a decreasing trend in burnout prevalence.

**Methods:**

Data collection took place in three waves using the computer assisted web interviewing (CAWI) method. In 2014 *n* = 1,027, in 2017 *n* = 1,024, and in 2020 *n* = 1,000. Respondents were selected from the European National Panel. Because the target group was adults (18–65 years), an online survey was chosen. Internet penetration in this target population is sufficient, and it was not necessary to use a combination of methodologies.

**Results:**

The results suggest a decreasing tendency for burnout syndrome in the Czech Republic. Knowledge about burnout syndrome is increasing, suggesting that people are paying more attention to their mental health and the possible factors that might affect the severity of burnout syndrome.

**Conclusion:**

Overall, burnout threatens the general population, not only medical employees. It is a positive finding that over the years, there has been an increasing trend in awareness about burnout. It further shows that people have adopted certain precautions and steps to avoid burnout syndrome as the perceived threat from it gradually decreases.

## Introduction

Burnout syndrome is a state of long-term work exhaustion that manifests on three levels: cognitive, physical and emotional (1). Research regarding burnout syndrome has spiked in recent years. Despite burnout syndrome not being a clinical diagnosis, it has been recognized as a significant reason for work absence or, in some cases, even work leave (2). The predominant focus of burnout research has been and remains on medical personnel (3). However, burnout syndrome is no longer seen as a syndrome in the medical profession (4). In addition, there has been research on burnout among students and teachers (5).

The ever-accelerating pace and increased work stress are reasons for the growing burnout rates. A longitudinal study from China points to a deteriorating world “climate,” which leads to increasing burnout and depression. The results also suggest varying degrees of association between work environment, depression and burnout (6). Therefore, we hypothesize that depression and burnout are closely related but not identical concepts. Burnout is strongly associated with a sense of work-life balance; the individual expects the energy invested in work to be restored. Thus, it is closely linked to the perception of equity at work (7) and the support that an individual receives from their supervisors and coworkers (8, 9).

Sociodemographic factors have also been extensively studied. However, the obtained results are not unambiguous. Regarding age, some studies have concluded that burnout syndrome increases with age (10, 11), while others report higher levels of burnout in younger populations (12). Work stress and lack of time to adjust can be significant causes of burnout in young individuals.

Furthermore, Maslach and Jackson stated that sex alone is not a critical determinant of burnout (13). However, a recent study revealed specific differences in burnout syndrome, with women reporting more emotional exhaustion and men reporting more depersonalization (14).

In the Czech Republic, burnout among medical professionals has been studied and documented. Research has also been published regarding burnout among teachers (15–17). However, there was not a study that would focus on general population. Previous studies only focused on specific sub-group and therefore a comprehensive study reporting the prevalence of burnout among the general adult population in the Czech Republic is lacking. This study tries to fill in this gap. As literature suggest burnout syndrome is a comprehensive syndrome that affects population regardless of their job (or field of studies) and therefore we did not examine any specific sub-groups. This study examines trends in burnout in the Czech population. The main aim of this research was to fill in the burnout literature gap in the Czech Republic. and document the burnout trend over the years. Our secondary aim was to see if there is awareness regarding burnout syndrome and whether, over the years, we will see an increasing or a decreasing trend in burnout prevalence.

## Methodology

Data were obtained in cooperation with the STEM/MARK agency and respondents from the European National Panel. We opted for the computer assisted web interviewing method of online questioning. This method is well justified since we were aiming for the adult population, and we know from previous census studies that internet penetration is high among this population. It is a fast and reliable way of collecting data and reaching larger number of respondents. Due to a quota based on sex, age, education, and region inhabited, a representative sample of the Czech people has been achieved. Data were collected at three time points: 2014, 2017, and 2020. Participation was voluntary, and participants were informed about their right to withdraw at any time during the questionnaire.

For the measurement of burnout, we chose the Shirom Melamed Burnout Measure. This measure has been translated into the Czech language, and a study of its validity and reliability has shown that it has appropriate psychometric properties with the overall Cronabach Alpha reaching 0.94 and the three subscales also showed good internal consistency. Confirmatory factor analysis showed 3 factors – emotional, cognitive and physical burnout (18). The total score per individual was obtained based on the sum of multiple Likert items. This new variable was considered approximately continuous (or at least approximated to an interval scale). Furthermore, norms for the Czech population have been calculated and are accessible online.^1^ Data were analyzed in STATA software version 17.

## Results

In 2014, we collected data from 1,027 respondents (52.78% men, 47.22% women); in 2017, *n* = 1,024 (52.73% men and 47.27% women); and in 2020, *n* = 1,000 (49.4% men and 50.6% women). According to the Czech Statistical Office, there are more women (50.8%) than men (49.2%) in the Czech population. However, there are more men (51.2%) than women (48.8%) in the 15–59 age group. In the 2014 sample, 52.78% were men and 47.22% were women. In the 2017 sample, 52.73% were men and 47.27% were women. This distribution corresponds to the ratios given by the Czech Statistical Office, where even in our sample there were more men than women. In 2020, there were fewer men than women, yet the sample was evenly distributed, with 49.4% men and 50.6% women. The number of respondents and the distribution between sexes make these 3 data collections comparable.

The second sociodemographic variable examined was age. The average age of the Czech population in 2014 was 41.7 years. For women, the average age was slightly higher (43.3) than for men (40.2). In 2017, the average age was 42.2 years, again higher for women (43.6) than for men (40.8). The latest available data is from 2018, when we again observe an increase in the average age to 42.3 years, and similarly to previous years, the average age for women is higher (43.7) than for men (40.9). The average age of the Czech population is steadily increasing and, similarly to other developed countries, the average age of women is higher than that of men. We looked whether our data also corresponded to this. The average age of men and women during all three data collection periods was comparable. The standard deviation indicates a large spread in the data, which is unsurprising as the population aged 25–65 was sampled (even 18–65 in the last year of data collection). During the first two data collections, men were older than women, in 2020 women were slightly older than men. The lower average age in the last year of data collection is because the group of 18–25 years old was included among the respondents so that the collected data corresponds to the age of the adult population in the Czech Republic. According to the definition of the United Nations, the population 65+ can already be described as “old age.” In the Czech Republic, the age of 65 is also referred to as the highest retirement age, and therefore the decision was made not to include the older population.

The last sociodemographic variable was education. The category of education was divided into standard categories corresponding to the education system in the Czech Republic: elementary (primary), secondary school (secondary) - with or without a high school diploma (learning certificate), and university (tertiary). According to the Czech Statistical Office, the percentage of the population with higher education is increasing, and depending on the age of the population, either secondary education without a high school diploma or with a high school diploma prevails. Overall, it is evident from the data that collected data are comparable populations in terms of gender, age and education, and the data collection methodology ensured a representative sample of the Czech population. Gender, as well as age and education, were evenly distributed and corresponded to data provided by the Czech Statistical Office on the population.

In terms of analysis as a first variable, we were interested in the awareness of burnout syndrome (Table 1).

**Table 1:** Knowledge of burnout syndrome.

Are you familiar with the term burnout syndrome?						
	2014		2017		2020		*n*	%	*n*	%	*n*	%
Yes	804	78.29	846	82.62	862	86.2
No	223	21.71	178	17.38	138	13.8

We observed that over the years, knowledge about burnout syndrome has risen by almost 8%. Therefore, more people currently report that they know the term burnout syndrome. Furthermore, we explored whether they felt subjectively threatened by burnout syndrome.

In Table 2, we observed a decreasing trend in that area, as fewer people felt threatened by burnout syndrome. It can be assumed that increasing knowledge of the syndrome increases knowledge about possible prevention and intervention. Thus, the threat of the syndrome decreased as it is not something unknown anymore. The subjective feeling of being threatened by burnout syndrome was significantly correlated with the actual burnout levels measured by the Shirom Melamed Burnout Measure. When looking at burnout alone, each year, we saw significant differences among the sexes, with women reporting considerably significantly higher burnout levels than men. Overall, the total burnout levels were also decreasing (Table 3).

**Table 2:** Subjective threat of burnout syndrome.

Do you feel threatened by the burnout syndrome?						
	2014		2017		2020		*n*	%	*n*	%	*n*	%
Definitely yes	87	8.47	67	6.54	63	6.3
More likely yes	242	23.56	224	21.88	186	18.6
More likely no	389	37.88	390	38.09	395	39.5
Definitely no	219	21.32	211	20.61	255	25.5
I do not know	90	8.76	132	12.89	101	10.1

**Table 3:** Burnout levels.

Burnout						
Year	Total			Men			Women			x̅	St. Dev.	Coefficients of variation	x̅	St. Dev.	Coefficients of variation	x̅	St. Dev.	Coefficients of variation
2014	42.24	16.22	38.40	40.83	15.68	38.40	43.81	16.69	38.10
2017	40.19	15.53	38.64	38.67	15.62	40.39	41.88	15.26	36.44
2020	39.12	15.7	40.13	37.72	14.96	39.66	40.49	16.3	40.26

For the overall sample, Bartlett’s equal-variances test showed that: chi^2^ = 2.12 (*p* = 0.345) and thus, Analysis of variance (ANOVA) was utilized to see whether the differences in burnout between years are statistically significant. ANOVA confirmed these differences were statistically significant *F* (2, 3,048) = 10.21, *p* < 0.001. Subsequent Bonferroni analysis showed that the significant differences were between the years 2014 and 2017 (*p* = 0.01) and between the years 2014 and 2020 (*p* < 0.001). The effect size measure η2 for this case indicates that our model accounts for approximately 0.6% of the variability in SMBM scores and the 95% confidence interval does not include the null value of zero (0.18, 1.31%).

For the next step of the analysis, the sample was split into two according to the sexes of the respondents. For males, Bartlett’s equal-variances test showed that chi^2^ = 1.36, *p* = 0.50, meaning we do not reject the null hypothesis for the equality of variance and analysis of variance (ANOVA) was run to explore the differences between the burnout levels for different years. The differences in burnout for males were statistically different between years *F* (2, 1,573) = 5.6, *p* = 0.0038. Post-hoc Bonferroni test showed that the significant difference was only between 2014 and 2020 (*p* = 0.004). The η^2^ indicates that our model accounts for approximately 0.7% of the variability in SMBM scores, and the 95% confidence interval does not include the null value of zero (0.08, 1.68%). This value suggests a weak effect size.

For females, Bartlett’s equal-variances test showed chi^2^ = 4.08, *p* = 0.13. Similarly, to males, we do not reject the null hypothesis for the equality of variance, and ANOVA was performed. This model was also significant as *F* (2, 1,472) = 5.31, *p* = 0.005, and the significant differences were between 2014 and 2020 (*p* = 0.004). The overall η2 indicates that our model accounts for approximately 0.71% of the variability in SMBM scores, and the 95% confidence interval does not include the null value of zero (0.07, 1.73%). This also showed a significant yet weak effect size.

With this relevant decrease trend over the years, we wanted to see whether the norms established in 2014 are still applicable even today. Therefore, we transferred the raw burnout scores according to the previously set norms for the Czech population.

Table 4 results suggest that although the norms are still appropriate and the majority of the Czech population falls within one standard deviation range (which corresponds to the normal distribution), if this observation of a decreasing trend continues, norms will have to be reconsidered and adjusted.

**Table 4:** Burnout according to norms.

	Total		Men		Women		*n*	%	*n*	%	*n*	%
2014						
−2 std. dev.	168	16.36	83	15.31	85	17.53
−/+1 std. dev.	680	66.21	367	67.71	313	64.54
+2 std. dev.	148	14.41	71	13.10	77	15.88
+3 std. dev.	27	2.63	18	3.32	9	1.86
+4 std. dev.	4	0.39	3	0.55	1	0.21
2017						
−2 std. dev.	205	20.02	117	21.67	88	18.18
−/+1 std. dev.	680	66.41	348	64.44	332	68.60
+2 std. dev.	116	11.33	61	11.30	55	11.36
+3 std. dev.	19	1.86	10	1.85	9	1.86
+4 std. dev.	3	0.29	3	0.56		
+5 std. dev.	1	0.10	1	0.19		
2020						
−2 std. dev.	233	23.3	114	23.08	119	23.52
−/+1 std. dev.	646	64.6	317	64.17	329	65.02
+2 std. dev.	93	9.3	47	9.51	46	9.09
+3. std. dev.	19	1.9	10	2.02	9	1.78
+4 std. dev.	9	0.9	6	1.21	3	0.59

## Discussion

Our results suggest a decreasing tendency for burnout syndrome in the Czech Republic. Knowledge about burnout syndrome is increasing, meaning that people pay more attention to their mental health and the possible factors that might affect the severity of burnout syndrome. This also corresponds with the decreasing subjective threat from burnout syndrome. The subjective feeling of being threatened by burnout syndrome significantly correlated with the Shirom Melamed Burnout Measure results. What we perceive as a very positive result is that over the years, we have seen a statistically significant decrease in the levels of burnout. We believe people took more preventive measures with increased awareness about this potential problem.

As the knowledge of the population regarding burnout increases, they are also more aware of the possible preventions and interventions; thus, burnout does not pose such a threat anymore. This result is similar to what has been observed in previous studies on the teacher population (5). However, we need to be mindful that the effect sizes in our results were relatively small, suggesting that although there is an increased level of knowledge regarding burnout syndrome, not all people act on the knowledge they acquire. Thus, it remains essential to keep raising the awareness and information level regarding burnout.

As previous studies reported, this research also clearly shows that sex is crucial when studying burnout. Women reported significantly higher levels than men did. This corresponds to results found by for example (3, 13, 19, 20). Furthermore, our study is in congruence with studies reporting specific subtypes of burnout for women (14).

This research also supports the argument that burnout syndrome is in fact a stand-alone category and not just a subtype of depression. As we can see it is a syndrome affecting the overall and general population. Lastly, this research has clearly shown that burnout syndrome is a syndrome that is independent of the profession and that it can be a concern for all working adults (4) and not only of those working in the helping or medical professions.

A specific limitation of this study is that although the data were collected during 3 different years with the same national panel, it has not always been the same respondents. Therefore, the data we present are data from 3 cross-sectional studies. However, for all three timepoints, we collected a representative sample of the Czech population; therefore, we believe that the claim that burnout syndrome is periodically decreasing is justifiable. Another limitation of this study is that the first wave of data collection occurred during autumn and the second and third waves occurred during spring. Therefore, the time when the data were collected might have impacted the current burnout situation. However, we also observed a decrease from 2017 to 2020, when both data sets were collected during the same time of the year.

## Conclusion

Overall, burnout threatens the general population, not only medical employees. It is a very positive observation that over the years, there has been increasing knowledge about burnout. It also shows that people have adopted certain precautions and steps to avoid burnout syndrome as the perceived threat from it gradually decreases. This means we need to actively continue in our effort to educate the population about the importance of mental health and mental well-being. In future research, it would be interesting to see how burnout changes over the years and whether it is similar to certain seasonal affective disorders in that it changes dramatically within the scope of a year.

If this decreasing trend continues, the norms set for the Czech population in 2013 will have to be revisited and readjusted shortly, as the mean level of burnout is now considerably lower.

To our knowledge, this is the first study to observe the levels of burnout over a more extended period in the Czech Republic. This study brings new and unique knowledge by clearly demonstrating how burnout levels decrease and are associated with increased knowledge of burnout syndrome.

In the future, this research could be enriched by studying the reported knowledge about burnout syndrome more in-depth and investigating whether individuals are taking active precautions against burnout syndrome. Furthermore, we have shown that it affects the general population, so specific sub-groups can be studied more. As burnout syndrome affects students and working individuals, it could be interesting to compare different types of employment or other types of schools. Lastly, we recommend that future studies update the norms we currently have in the Czech population. As our research has shown the decreasing trend in the syndrome, we expect the norms to be soon outdated.

## Data availability statement

The datasets presented in this article are not readily available because participants were informed their data will be used by research from First Faculty of Medicine only. Requests to access the datasets should be directed to martina.vnukova@lf1.cuni.cz.

## Ethics statement

The studies involving human participants were reviewed and approved by General university hospital ethics committee. The patients/participants provided their written informed consent to participate in this study.

## Author contributions

IS performed the literature search and helped write the manuscript. MSV performed the editing and statistical analysis of the manuscript and helped write the manuscript. TB performed the literature search. MA helped write the manuscript and provided financial support. RP helped write the manuscript. All authors contributed to the article and approved the submitted version.

## Funding

This work was supported by the Cooperatio Program, research area Psychology and Cooperation Neurosciences.

## Conflict of interest

The authors declare that the research was conducted in the absence of any commercial or financial relationships that could be construed as a potential conflict of interest.

## Publisher’s note

All claims expressed in this article are solely those of the authors and do not necessarily represent those of their affiliated organizations, or those of the publisher, the editors and the reviewers. Any product that may be evaluated in this article, or claim that may be made by its manufacturer, is not guaranteed or endorsed by the publisher.

## Footnotes

^1^ https://psychiatrie.lf1.cuni.cz/supplementary-data
